# Altered Phenotypic Responses of Asexual Arctic *Daphnia* After 10 Years of Rapid Climate Change

**DOI:** 10.1111/gcb.70119

**Published:** 2025-03-18

**Authors:** Athina Karapli‐Petritsopoulou, Jasmin Josephine Heckelmann, Dörthe Becker, N. John Anderson, Dagmar Frisch

**Affiliations:** ^1^ Department of Evolutionary and Integrative Ecology Leibniz Institute of Freshwater Ecology and Inland Fisheries (IGB) Berlin Germany; ^2^ Department of Biology, Chemistry, Pharmacy, Institute of Biology Freie Universität Berlin Berlin Germany; ^3^ NABU‐Naturschutzstation Niederrhein Kleve Germany; ^4^ Department of Geography and Environment Loughborough University Loughborough UK

**Keywords:** critical oxygen limit, environmental change, freshwater, hypoxia tolerance, phenotypic adaptation, respiration rate, resurrection ecology, thermal tolerance, whole genome sequencing, zooplankton

## Abstract

Understanding the fates of organisms and ecosystems under global change requires consideration of the organisms' rapid adaptation potential. In the Arctic, the recent temperature increase strongly impacts freshwater ecosystems which are important sentinels for climate change. However, a mechanistic understanding of the adaptive capacity of their key zooplankton grazers, among them polyploid, obligate parthenogenetic *Daphnia*, is lacking. Theory suggests low adaptation potential of asexual animals, yet examples exist of asexuals persisting through marked environmental changes. Here, we studied asexual *Daphnia pulicaria* from a meromictic lake in South‐West Greenland. Its oxycline hosts purple sulfur bacteria (PSB), a potential food source for *Daphnia*. We tested two key phenotypic traits: (1) thermal tolerance as a response to rapid regional warming and (2) hypoxia tolerance tied to grazing of PSB in the hypoxic/anoxic transition zone. To assess *Daphnia*'s adaptive capacity, we resurrected *Daphnia* from dormant eggs representing a historical subpopulation from 2011, sampled modern subpopulation representatives in 2022, and measured phenotypic variation of thermal (time to immobilization—*T*_imm_) and hypoxia tolerance (respiration rate and critical oxygen limit—*P*_crit_) in clonal lineages of both subpopulations. Whole genome sequencing of the tested clonal lineages identified three closely related genetic clusters, one with clones from both subpopulations and two unique to each subpopulation. We observed significantly lower *T*_imm_ and a trend for higher *P*_crit_ and respiration rates in the modern subpopulation, indicating a lower tolerance to both high temperature and hypoxia in comparison with the historical subpopulation. As these two traits share common physiological mechanisms, the observed phenotypic divergence might be driven by a relaxed selection pressure on hypoxia tolerance linked to variation in PSB abundance. Our results, while contrary to our expectation of higher thermal tolerance in the modern subpopulation, provide evidence for phenotypic change within a decade in this asexual *Daphnia* population.

## Graphical abstract

Freshwater ecosystems in the Arctic are important sentinels for climate change, but not much is known about the potential for rapid adaptation of their asexually reproducing key zooplankton member. We compared thermal and hypoxia tolerance of resurrected dormant stages of *Daphnia* from an Arctic meromictic lake to that of their modern descendants. The detected physiological and genetic differences are potentially linked to the absence of anoxic microbial populations. A destabilization of these microbial populations may have been caused by more frequent events of early ice‐out in the past decade resulting from the changing climate.

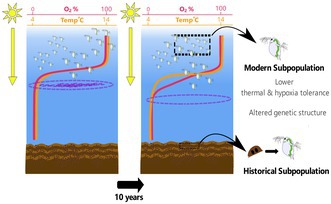

## 1 Introduction

Environmental change is a powerful driver of selection and evolutionary adaptation (MacColl 2011; Garant 2020). However, if changes occur too rapidly, the microevolutionary capacity of organisms may fail to track them (Bürger and Lynch 1995; Chevin et al. 2010). Under the current unprecedented rate of environmental change (IPCC 2023) it is unclear which organisms will persist and under which conditions, as ecosystems are being affected globally (Parmesan 2006; Finn et al. 2023). It is therefore crucial to understand whether and how organisms can adapt at pace with the rapidly changing environments (Visser 2008; Hoffmann and Sgrò 2011).

Growing evidence highlights the significant potential of sexually reproducing organisms for rapid adaptation to climate change (Bradshaw and Holzapfel 2006; Catullo et al. 2019). However, the rapid adaptive capacity of asexual natural populations is still poorly understood (Bast 2018; Jaron et al. 2021). Evolutionary adaptation is facilitated by the process of natural selection acting upon genetic variation (Agashe et al. 2023). To generate this variation, sexual organisms benefit from genetic recombination, a major force in the evolution and establishment of sexual reproduction (Crow 1994). Spontaneous mutations additionally contribute to genetic variation, but are expected to produce mainly deleterious effects, which should constrain the mutation rate at an evolutionary low optimum (Kimura 1967). Without the ability for recombination, asexual lineages are thus bound to accumulate nonbeneficial mutations in the long term (known as Müller's ratchet), resulting in mutational meltdown (Lynch et al. 1993). Conversely, recent studies of vertebrate and invertebrate asexuals did not find evidence for a reduced fitness of long‐lived clones compared with sexual congeners despite the accumulation of mutations (Kočí 2020; Kearney et al. 2022) and uncovered that the asexual genome is much more dynamic than previously thought, including high rates of gene conversion, ameiotic recombination, and deletions (Xu et al. 2011; Tucker et al. 2013; Jaron et al. 2021).

The microcrustacean *Daphnia*, a freshwater keystone grazer, is an important model for rapid evolution. Species of this genus generally reproduce by cyclical parthenogenesis (CP), but obligate parthenogenesis (OP) exists, and in higher latitudes is often coupled with polyploidy (Weider 1987; Dufresne and Hebert 1997; Decaestecker et al. 2009). Many studies have evidenced the genetic and phenotypic responses of CP *Daphnia* over centuries and even decades to cultural eutrophication (Frisch et al. 2014), dietary cyanobacteria (Hairston 1999), predation pressure (Cousyn et al. 2001; Chaturvedi 2021), parasites (Decaestecker et al. 2007), salinization (Wersebe and Weider 2023), and warming (Geerts 2015; Yousey et al. 2018). However, studies in OP *Daphnia* that address the question of rapid adaptation are lacking, especially in Arctic regions where climate change has particularly severe effects.

A powerful method to directly observe evolutionary change in a population across different time periods is known as resurrection ecology. It uses revived dormant stages from natural populations archived in lake sediment (Burge et al. 2018; Weider et al. 2018) for the experimental study of otherwise inaccessible phenotypic traits of ancestral populations.

South‐West Greenland is a suitable region to test potential contemporary microevolutionary responses, as it has been undergoing particularly pronounced environmental change during the last decades. Average June air temperatures in this region, representing the end of spring thaw, have increased by 2.2°C in the last 30 years, while average summer temperatures (July) increased by 1.1°C in the last 20 years (Saros 2019). Associated with this average increase of air temperature, there is a trend towards an earlier lake ice‐out (Šmejkalová et al. 2016). A recent study in the region of Kangerlussuaq, SW Greenland, showed that the timing of ice‐out has implications for the duration of spring mixing and oxygenation of the water column (Hazuková et al. 2024). This area also harbors several oligosaline meromictic lakes (Anderson et al. 2001). These oligotrophic, fishless lakes contain Arctic *Daphnia pulicaria* , a member of the *Daphnia pulex* species complex (Colbourne et al. 1998). Following the pattern often observed toward higher latitudes (Beaton and Hebert 1988), *Daphnia* populations in this area are polyploid and obligate parthenogens (asexuals) (Dane et al. 2020).

We focused our study on one of these lakes, Braya Sø, where a single asexual clone has dominated the population for at least the past 200 years (Dane et al. 2020), suggesting a genetically nearly uniform population. An important characteristic of this lake is the presence of phototrophic purple sulfur bacteria (PSB) that form a bacterial plate in the upper part of the anoxic zone. The sediment record indicates that the abundance of PSB is dynamic over time, with periods of near absence (McGowan et al. 2008), and that their abundance is correlated with the abundance of ephippia, the dormant stages of *Daphnia* (Anderson et al. submitted). PSB are abundant in this region and are present in at least eight lakes in the near vicinity of Braya, observed either through direct sampling or pigment analysis; most of these lakes are also inhabited by *Daphnia* (Anderson unpublished data; McGowan et al. 2008; Reuss et al. 2013). Studies have shown that anaerobic bacteria communities can contribute significantly to crustacean zooplankton diets (Overmann et al. 1999; Kankaala et al. 2010). Moreover, *Daphnia* could be relying on PSB as either a direct or an indirect food source (Massana et al. 1994). To exploit PSB, *Daphnia* require tolerance to hypoxia to allow them to graze in or near the anoxic zone at least for short periods of time. Hypoxia tolerance could also have implications for thermal tolerance as both traits are strongly associated with the efficiency of oxygen metabolism (Pörtner 2002).

In this study, we asked whether phenotypic evolution in an asexual population is fast enough to track rapid environmental change. Given the recent temperature increase in the area, we tested the possible adaptation of modern clones to higher temperatures. We assessed thermal tolerance by measuring time to immobilization (*T*_imm_) of multiple clonal lineages of two *Daphnia* temporal subpopulations (modern and historical) from Braya Sø. To assess the genotypic variation present in the two temporal subpopulations, we performed whole genome sequencing. Members of the current Braya Sø population had lower respiration rates than populations in neighboring holomictic lakes (Karapli‐Petritsopoulou et al. 2024). To further explore hypoxia tolerance in Braya Sø, we measured respiration rates and determined the critical oxygen limit (*P*_crit_) in members of both temporal subpopulations. Respiration rate (i.e., oxygen consumption) was expected to be low in this population to allow feeding on PSB in the hypoxic to anoxic zone (Seidl et al. 2005; Karapli‐Petritsopoulou et al. 2024). Similarly, a low *P*_crit_ would signal high hypoxia tolerance.

## 2 Methods

### 2.1 Environmental Data

To determine whether there has been a change in summer water temperature at Braya Sø (Lake code: SS4; 66°59′24.1″ N 51°01′39.8″ W) over the study period, we used the known relationship between ice‐out date (IoD; as day of the year) and mean July water temperature (determined by in situ data loggers; see Anderson and Brodersen 2001: mean July water temperature = −0.1018 × [IoD] + 3820.4; *r* ^2^: 0.641; *p* < 0.01). In the Kangerlussuaq area, regional surveys have shown that mean May air temperature [May‐T] determines ice‐out date: IoD = −2.33 × [May‐T] + 165.24; *r* ^2^ = 0.797; *p* < 0.05. May air temperature was taken for the years 2005–2016 from the DMI meteorological station at Kangerlussuaq airport. For the period 2000–2004 (*n* = 5) ice‐out date was inferred from in situ data loggers (measuring at either 1 or 2 hourly intervals) confirmed by remote automatic cameras (see Anderson and Brodersen 2001). For the period 2017–2023, ice‐out was determined using satellite imagery (Sentinel‐2 which has a 2‐day return frequency at this latitude [66°N]); it was not possible to determine ice‐out for the lake in 2022 due to cloud cover during the relevant time period. Between 2000 and 2020, ice‐out date varied by 29 days (minimum day 141; maximum day 170) which gives an inferred mean July water temperature range of 11.04°C–13.77°C.

### 2.2 Field Sampling

Sediment from Braya Sø, South‐West Greenland was sampled in April 2022 by extracting three sediment cores with a Hon‐Kajak sediment corer (diameter 9 cm), from the deepest part of the lake, 1 m apart from each other. Sediment cores were sectioned in the field (0.5 cm interval) and kept at 4°C until processing. In July 2022, we sampled adult *Daphnia* from the entire water column using a 200 μm plankton net (diameter 25 cm). Temperature and oxygen profiles of the water column were measured on the day of *Daphnia* sampling (YSI 650 MDS multiprobe). In previous years, a purple sulfur bacteria population was present near the oxycline between 9 and 14 m depth (Anderson unpublished data). After determining the depth of the oxycline, we sampled the water column at multiple locations in 50 cm increments between 9 and 14 m, using a 5 L Van‐Dorn bottle (height 41.5 cm, diameter: 13 cm), but were unable to detect the PSB population.

### 2.3 Sediment Core Dating

Radiometric dating of the core was performed at the St. Croix Watershed Research Station, Minnesota, USA, following the method for Lead‐210 dating described in Appleby (2001). Results are shown in Table S1.

### 2.4 Resurrection of Ephippia From the Sediment

*Daphnia* ephippia were removed from the sediment and decapsulated using watchmaker's forceps. Eggs from individual ephippia were separated into 2 mL SSS medium (Saebelfeld et al. 2017). Hatching conditions were 10°C and a photoperiod of 18:6 light:dark. The resurrected clones used in this study were all hatched from eggs retrieved from the 1–1.5 cm sediment section, which was dated to be from 2011 (Table S1). Once hatched, juveniles were transferred to 100 mL jars with SSS medium at 14°C and 24 h dimmed light.

### 2.5 Clonal Cultures

The clones used in this study were each established from one *Daphnia* female cultured in an incubator at 14°C under 24 h dimmed light for at least 6 months before the start of measurements, equivalent to ca. nine generations. A water temperature of 14°C is regularly reached in the upper water column during the warmest summer months. They were fed with 1 mg C L^−1^ of *Tetradesmus obliquus* (Turpin) M.J. Wynne, 2016 three times a week. For the experiments of this study, we used 11 clones representing a historical subpopulation resurrected from a sediment section dated to 2011 and 12 clones representing the modern subpopulation (sampled in summer 2022).

### 2.6 Whole Genome Sequencing

Genomic DNA extraction from all 11 historical and 12 modern clones was performed using the MasterPure Complete DNA and RNA Purification kit (Biozym) according to the manufacturer's instructions. Before DNA extraction, *Daphnia* were treated with a mixture of antibiotics (50 mg L^−1^ ampicillin and 50 mg L^−1^ tetracycline dissolved in SSS medium) and fed with Sephadex G‐50 beads (5 g L^−1^) for 2 days prior to DNA extraction. Paired‐end (PE) sequencing libraries were prepared with the Illumina DNA Prep PCR‐free method (~300 bp insert size). Sequencing was done on an Illumina NovaSeq 6000 S4 Flowcell with 150 bp paired‐end reads and an average of 6 Gb per sample. Library preparation and sequencing were performed at the Competence Centre for Genomic Analysis (CCGA) in Kiel, Germany. Fastq files of DNA sequences included here are available at GenBank under Bioproject PRJNA1147697 with accession numbers SRR32522081—SRR32522099 and SRR30223773—SRR30223776.

### 2.7 Bioinformatic Analyses

Following O'Grady et al. (2022), we used FASTQC 0.11.9 (Andrews 2015) for quality control of PE reads and TrimGalore 0.6.6 (Krueger et al. 2023) for adapter trimming with a Phred score cutoff of 20 and discarding paired reads shorter than 35 bp after trimming. Reads were mapped to the *Daphnia pulex* reference genome (NCBI accession GCF_021134715.1) with BWA‐MEM 0.7.17 (Li and Durbin 2009) and default parameters. Duplicate and supplementary reads were removed with *markduplicates* (Picard Toolkit 2.26; Broad Institute 2024) and *samtools view* (Samtools 1.10; Danecek 2021). Single nucleotide polymorphisms (SNPs) were called using *freebayes* 1.3.2 (Garrison and Marth 2012) with a ploidy setting of 3, and excluding regions with extremely high coverage (‐g 10,000). For this variant call, only alignments with a mapping quality > 40 and alleles with a base quality > 24 were included. The options—min‐alternate‐fraction and—min‐alternate‐count were set to 0.05 and 5, respectively. Next, we filtered variants with vcffilter from vsflib 1.0.3 (Garrison et al. 2022) using the parameters QUAL > 1, QUAL/AO > 10, SAF > 0 and SAR > 0, RPR > 1, and RPL > 1. The above analyses were conducted within the high‐performance computing infrastructure at ZEDAT, Freie Universität Berlin (Bennett et al. 2020).

The last stage of filtering and further analysis was performed in R (R Core Team 2023). To obtain the final SNP set, we selected polymorphic, biallelic SNPs with a minor allele frequency (MAF) of 0.15 and an average read depth per sample of 10–1000 reads using the R package *SeqArray* 1.26.2 (Zheng et al. 2017). From this set, we calculated pairwise genome‐wide identity‐by‐state (IBS) similarity with the *snpgdsIBS()* function of the SeqArray package modified for triploid genomes (details in Karapli‐Petritsopoulou et al. 2024). The package *SNPrelate* (Zheng et al. 2012) was then used to compute the hierarchical cluster analysis. Principal component analysis (PCA) of SNPs was computed with the package *smartsnp* (Herrando‐Pérez et al. 2021).

### 2.8 Time to Immobilization

*Daphnia* of the 23 clones described above were used for time to immobilization (*T*_imm_) tests. These were conducted in two batches with two experimental runs each. Each batch contained a mixture of randomly chosen clones from both subpopulations. Each run had three replicates of each clone from the respective batch (six replicates per clone in total). *T*_imm_ was measured on preadults, aged 10–18 days.

#### 2.8.1 Measurement Setup

To measure *T*_imm_, we used a custom‐made apparatus following the design from Burton et al. (2018). The apparatus consisted of 45 6 mL vials with a 1.6 cm diameter and a 4 cm height that were fitted in a plexiglass box of 28.5 cm × 17.5 cm × 5.5 cm in dimensions in five rows of 9 vials. The box was sealed with silicone and connected to a refrigerated water circulator through two main openings as inflow and outflow on two sides. The two openings were separated into five smaller ones per side, placed between the rows of vials to allow for optimal water flow and temperature control.

#### 2.8.2 Determination of CT_Max_

To establish the temperature of the *T*_imm_ assay, we first determined the average CT_Max_ in mixed clone preadults from Braya Sø. For this test, we used the apparatus described above connected to the refrigerated water circulator. *Daphnia* were placed individually in each vial and were observed throughout the trial. Starting from 18°C, we increased the water temperature by 1°C every 6 min. The CT_Max_ was reached when all *Daphnia* exhibited immobilization due to thermal stress and was determined to be 36°C for the Braya Sø *Daphnia*.

#### 2.8.3 *T*_imm_ Measurement

We measured *T*_imm_ on preadult *Daphnia* at 34°C (two degrees below CT_Max_), following Burton et al. (2018). *Daphnia* were placed individually into the vials in a randomized order, and the temperature was increased from 18°C to 34°C within 16 min to avoid a sudden heat shock. *Daphnia* swimming activity was recorded using a Sony Alpha AIII camera with a Tamron HA036 objective mounted on a camera tripod (Hitchy, Hitchy Handy Stativ). A light table (HSK, A4 LIGHT PAD) was positioned beneath the plate to enhance visibility. After all *Daphnia* had reached *T*_imm_, they were removed from the apparatus and photographed under a dissecting scope (ZEISS, STEMI 508) at a magnification of 25× using the image analysis software MikroLive v.5 (https://www.mikroskopie.de/). Body length was measured from the top of the head to the apical base of the spine. *T*_imm_ of individual *Daphnia* was determined using the video material and defined as the moment when no movement was detected for at least 30 s.

### 2.9 Respirometry Setup

To estimate the respiration rate and the critical oxygen limit (*P*_crit_) of single *Daphnia*, we measured oxygen consumption at 18°C for 6 h using a closed respirometry system (Loligo Systems, Denmark) consisting of a 200 μL 24‐well plate fitted with oxygen sensor spots and an SDR SensorDish Reader (PreSens Precision Sensing GmbH, Germany). In a previous assay performed at 14°C, almost none of the *Daphnia* reached *P*_crit_ within a 6‐h period. Therefore, in this study, we applied a higher (18°C) measuring temperature to maximize the possibility of reaching *P*_crit_ in all *Daphnia* by raising their oxygen consumption. The plate was sealed with a PCR film. Before adding individual *Daphnia* to each well, they were filled with SSS medium. Air bubbles were removed from the wells, which were then topped up with medium before sealing the plate. To achieve a stable temperature, the 24‐well plate was immersed in a sealed flow‐through water bath (Loligo Systems, Denmark) connected to a circulating refrigerated system (circulator: Thermo Scientific HAAKE A10, thermostat: Thermo Scientific HAAKE SC100). The MicroResp software (Loligo Systems, Denmark) was used to conduct the measurements.

To account for the diffusion of oxygen through the PCR film, we measured oxygen differences at 18°C in a separate run without *Daphnia* over 4 h by placing air‐saturated medium in the wells and corrected the measurements with the diffusion rate (see below).

Due to the low fecundity and long time to maturation (aprox. 20 days) of *Daphnia* from the study lake, entering all clones in the measurements synchronously was not possible. We therefore performed the measurements within 6 weeks in two batches: one including all resurrected (historical) clones and one with the clones collected from the water column in 2022 (modern clones). To control for possible batch effects, the historical clone SS4‐5 was included in both batches as a reference. We used preadults of 11–20 days old for our measurements.

To measure *Daphnia* oxygen consumption, we separated each batch into six runs that were performed during six consecutive days. Each run included one replicate of each clone present in the batch, four replicates of the reference, and four blank wells containing medium from the *Daphnia* jars to account for microbial (background) respiration, yielding a total of six replicates per clone. The positions of the clones in the plate were randomized before each run using the Microresp software. After 6 h, we measured the body length of individual *Daphnia* as described above. In cases when a replicate was missing from a run due to handling, an extra replicate of the same clone was included in the next run.

### 2.10 Statistical Data Analysis

All statistical analyses were performed in R (v. 4.3.0; R Core Team 2023).

#### 2.10.1 *T*_imm_

*Daphnia* identified as males and female *Daphnia* with eggs were excluded from further analysis, as their different physiological states might have biased the results. Due to this, two clones from the historical subpopulation had to be excluded from further analysis because fewer than three replicates remained. Additionally, the reference clone SS4‐5 was randomly subsampled to six replicates to be included in the analysis. We estimated the dry weight from the body length of each *Daphnia* using the available Length‐Weight formula for this lake's population (Dry Weight = 9.015362 × Length^2.86448^) (Karapli‐Petritsopoulou et al. 2024). We fitted two sets of linear mixed models with ML (package lmerTest, v. 3.1‐3; Kuznetsova et al. 2017, based on lme4 v. 1.1‐33; Bates et al. 2015) and compared them within each set using a likelihood‐ratio test. The models included either genetic cluster or subpopulation as a fixed factor with the addition of weight in two of the models. The experimental run and clone nested either within genetic cluster or subpopulation were used as random factors:

Set 1:

model gen0: *T*_imm_ ~ 1 + (1|Run) + (1|Genetic Cluster:Clone)

model gen1: *T*_imm_ ~ Genetic Cluster + (1|Run) + (1|Genetic Cluster:Clone)

model gen2: *T*_imm_ ~ Genetic Cluster + Weight + (1|Run) + (1|Genetic Cluster:Clone)

Set 2:

model sub0: *T*_imm_ ~ 1 + (1|Run) + (1|Subpopulation:Clone)

model sub1: *T*_imm_ ~ Subpopulation + (1|Run) + (1|Subpopulation:Clone)

model sub2: *T*_imm_ ~ Subpopulation + Weight + (1|Run) + (1|Subpopulation:Clone)

The models with genetic cluster failed to converge due to the random effect of clone. We therefore removed the random effect to reduce complexity and reran Set 1 (i.e., model gen1: *T*_imm_ ~ Genetic Cluster + (1|Run)). The models picked by the likelihood‐ratio test were refitted with REML for reporting.

We used the package multcomp (v. 1.4‐25; Hothorn et al. 2008) to perform post hoc tests with the Holm method for genetic clusters. Data visualization was performed using the ggplot2 (v. 3.5.1; Wickham 2016) and patchwork (v. 1.2.0; Pedersen 2024) packages.

#### 2.10.2 Respiration Rate Calculation and Statistical Analysis

Rates of diffusion, background respiration, and *Daphnia* respiration were calculated with the respR package (v. 2.3.1; Harianto et al. 2019). The respiration rates were adjusted for the mean diffusion rate measured over 24 wells in 4 h and the background respiration of each run averaged over the blank wells. We estimated the dry weight of *Daphnia* based on their body length using the formula described above and adjusted the respiration rate by dividing by dry weight to obtain the weight‐specific respiration rate. To correct for the batch effect, we used the reference clone SS4‐5. For this, we calculated a bias weight as follows:

$$\text{Bias}=\frac{|{\bar{\mathrm{ref}}}_{\mathrm{run}}-\bar{\mathrm{ref}}|}{\bar{\mathrm{ref}}}$$

where ${\bar{\mathrm{ref}}}_{\mathrm{run}}$ is the mean mass‐specific respiration rate of the reference clone of a given run and $\bar{\mathrm{ref}}$ is the overall mean of the reference mass‐specific respiration rate. The bias was calculated separately for each of the six runs. To obtain standardized mass‐specific respiration rates (in the following Resp_standard_), we multiplied the mass‐specific respiration rate of each experimental *Daphnia* with the run‐specific bias. The reference clone was randomly subsampled to six replicates to be included in the analysis.

To assess whether genetic cluster or subpopulation were significant factors in explaining our data, we performed two model comparisons between a null model with only random effects and a full model containing either genetic cluster or subpopulation as a fixed effect. The random factors were experimental run and clone nested within subpopulation or genetic cluster, respectively. The models were fitted using the lmerTest (v. 3.1‐3; Kuznetsova et al. 2017) and the comparisons were done with the likelihood‐ratio test.

Set 1:

model gen0: Resp_standard_ ~ 1 + (1|Run) + (1|Genetic Cluster:Clone)

model gen: Resp_standard_ ~ Genetic Cluster + (1|Run) + (1|Genetic Cluster:Clone)

Set 2:

model sub0: Resp_standard_ ~ 1 + (1|Run) + (1|Subpopulation:Clone)

model sub: Resp_standard_ ~ Subpopulation + (1|Run) + (1|Subpopulation:Clone)

Data visualization was performed with ggplot2 (v. 3.5.1; Wickham 2016) and patchwork (v. 1.2.0; Pedersen 2024).

#### 2.10.3 *P*_crit_ Estimation and Statistical Analysis

*P*_crit_ was estimated using the broken stick method from the respR package (v. 2.3.1; Harianto et al. 2019). Many individuals did not reach *P*_crit_. To estimate the probability of reaching *P*_crit_ based on either subpopulation or genetic cluster membership, we used binomial generalized mixed models (glmer function; lmer package) and compared the null model with full models using the likelihood‐ratio test, following the same model structure as explained above for Sets 1 and 2, with the response variable (binom_*P*_crit_) coded as the success or failure of reaching *P*_crit_.

The unavailability of *P*_crit_ values in many test animals resulted in an unbalanced dataset between runs. Therefore, a linear mixed model approach to analyze *P*_crit_ differences was not possible. The *P*_crit_ of genetic clusters and subpopulations was compared with one‐way ANOVA and Welch's *t*‐test, respectively. Post hoc tests were done using the Holm method. We tested the relationship between weight‐adjusted respiration rates and *P*_crit_ with a linear regression (log(*P*_crit_) ~ log(weight_adj_rates)).

The datasets produced from the above measurements are available in Zenodo (Karapli‐Petritsopoulou et al. 2025).

## 3 Results

### 3.1 Environmental Data

**FIGURE 1:**
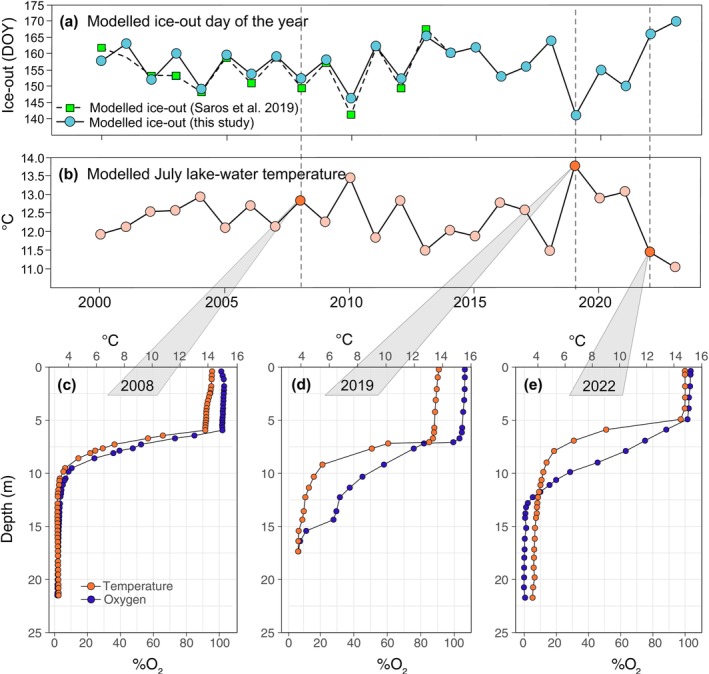
Change in ice‐out, temperature, and oxygen environment of Braya Sø during the study period. (a) Modelled lake ice‐out day of the year (DOY) estimated for this study is shown as blue circles from 2000 to 2023. For comparison, green squares with a dashed line show the ice‐out day estimated by Saros (2019). (b) Modelled average July surface lake water temperature. Dashed vertical lines in both (a) and (b) indicate the years 2008, 2019, and 2022, respectively. Depth profiles of August temperature (orange circles) and dissolved oxygen (DO % air saturation, blue circles) in Braya Sø measured in the years 2008 (c), 2019 (d) and 2022 (e).

The modelled lake ice‐out data show a pattern of increased variability after 2010, with both extreme early and late dates present (Figure 1a). Extreme early and late ice‐out dates reflect higher and lower modelled July surface lake water temperatures, respectively (Figure 1b). Exemplary August lake profiles from an average year before the increase in variability (2008) and 2 years after 2010, with an extremely early (2019) and an extremely late (2022) ice‐out date, show a deeper oxycline in both years after 2010, with 2019 being the deepest of the two (Figure 1d,e). The thermocline was at its deepest in 2019, followed by 2008 and 2022, matching the order from earlier to later ice‐out. Likewise, the upper mixed layer temperature was slightly reduced in August of 2019, the year with the earliest ice‐out, followed by 2008 and 2022.

### 3.2 Genomic Results

**FIGURE 2:**
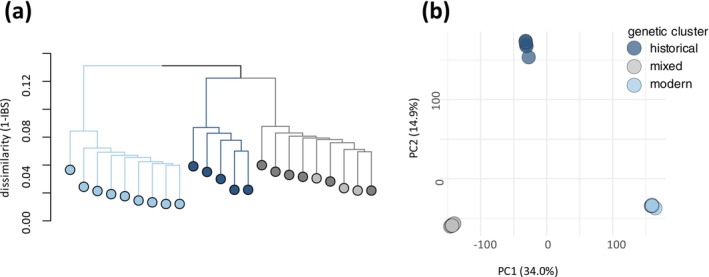
Genetic clusters of clonal lineages based on identity‐by‐state (IBS). (a) Dendrogram showing the results of hierarchical cluster analysis based on pairwise dissimilarity values (1‐IBS). From left to right the modern cluster is represented by light blue circles, the historical cluster by dark blue and the mixed cluster by gray circles. The darker and light gray shade in the mixed cluster represents clonal lineages from the historical and modern subpopulation, respectively. Each circle represents one clonal lineage. (b) Principal component analysis (PCA) of all identified SNPs, using the same color code as the dendrogram with one circle per clone.

A total of 365,466 SNPs remained after filtering and were used in further analysis. The hierarchical cluster analysis based on identity‐by‐state (IBS) identified three closely related genetic clusters: two clusters unique to each temporal subpopulation (a historical cluster with five of the 11 resurrected clones from 2011, and a modern cluster with nine clones sampled in 2022) and a mixed cluster that included six of the resurrected and three of the 12 modern clones (Figure 2a, Figure S1). The historical and mixed genetic clusters were more similar to each other, with a dissimilarity range (1‐IBS) of 8.75%–11.49%, while their common dissimilarity range to the modern cluster was 8.91%–13.33% (Table S2). These ranges are relative to the total number of SNPs identified. Based on an estimated genome size of 150 Mb, the highest dissimilarity of approximately 13% corresponds to a genome‐wide IBS of 0.03%. The PCA confirmed the clusters, with PCA1 separating mainly the mixed from the modern cluster and explaining 34% of the variation, while PCA2 separated the historical cluster from the other two and explained 14.9% of the variation (Figure 2b).

### 3.3 Time to Immobilization (*T*_imm_)

**FIGURE 3:**
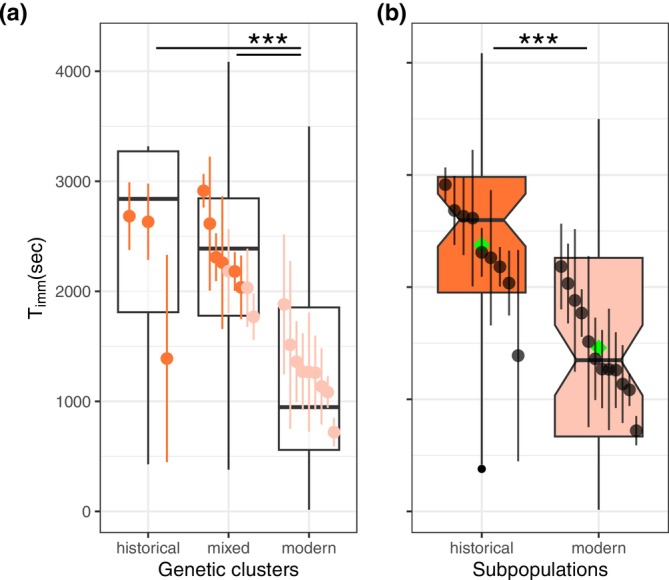
Time to immobilization (*T*_imm_) among genetic clusters (a) and between temporal subpopulations (b). The darker shades in both panels represent the historical subpopulation and the lighter shades the modern subpopulation. Point ranges in boxplots represent means per clone across replicates with SE. The boxplots are limited by the first and third quartiles, the middle line is the median and the whiskers are 1.5× interquartile ranges. Filled black circles at the bottom end of the whiskers represent outliers. In Panel b green diamonds indicate mean values per subpopulation. Horizontal bars above the boxes represent post hoc comparisons between genetic clusters (a, Holm method) and subpopulations (b, lmm model result). The statistical significance of the *p* value is shown by asterisks (***< 0.001). In Panel a both comparisons share the same statistical significance.

Both predictor variables “subpopulation” and “genetic cluster” explained the data variation significantly better than the null model, while the addition of “Weight” as a fixed factor did not improve the model (Table 1a). Time to immobilization (*T*_imm_) was significantly lower for the modern genetic cluster compared with the mixed and historical clusters (Figure 3a, Table 1b, Table S3). Additionally, the modern subpopulation showed a significantly lower *T*_imm_ compared with the historical subpopulation (Figure 3b, Table 1c).

**TABLE 1:** Model comparison with likelihood‐ratio test (a) and model results for model gen1 (b) and model sub1 (c) for time to immobilization (*T*_imm_).

a. Model comparisons	npar	AIC	BIC	logLik	Deviance	Chisq	df	p‐value
Set 1								
null: *T*_imm_ ~ 1 + (1|Run)	3	1869.76	1877.92	−931.88	1863.76			
gen1: *T*_imm_ ~ Genetic Cluster + (1|Run)	5	1830.32	1843.91	−910.16	1820.32	43.45	2	**3.68e‐10**
gen2: *T*_imm_ ~ Genetic Cluster + Weight + (1|Run)	6	1832.18	1848.49	−910.09	1820.18	0.13	1	0.715
Set 2								
null: *T*_imm_ ~ 1 + (1|Run) + (1|Subpopulation:Clone)	4	1855.03	1865.90	−923.51	1847.03			
sub1: *T*_imm_ ~ Subpopulation + (1|Run) + (1|Subpopulation:Clone)	5	1842.44	1856.03	−916.22	1832.44	14.59	1	**1.33e‐4**
sub2: *T*_imm_ ~ Subpopulation + Weight + (1|Run) + (1|Subpopulation:Clone)	6	1844.36	1860.67	−916.18	1832.36	0.07	1	0.787

b. Model gen1: *T*_imm_ ~ Genetic Cluster + (1|Run)					
	Estimate	Std. error	df	t value	p‐value
Intercept	2425.86	315.84	9.55	7.68	**2.19e‐5**
Genetic cluster mixed	−158.31	250.52	106.71	−0.63	0.529
Genetic cluster modern	−1271.07	255.65	106.97	−4.97	**2.54e‐6**

c. Model sub1: *T*_imm_ ~ Subpopulation + (1|Run) + (1|Subpopulation:Clone)					
	Estimate	Std. error	df	*t* value	p‐value
Intercept	2361.71	253.45	5.39	9.32	**1.59e‐4**
Subpopulation modern	−937.63	208.54	18.09	−4.50	**2.77e‐4**

### 3.4 Respiration Rate

**FIGURE 4:**
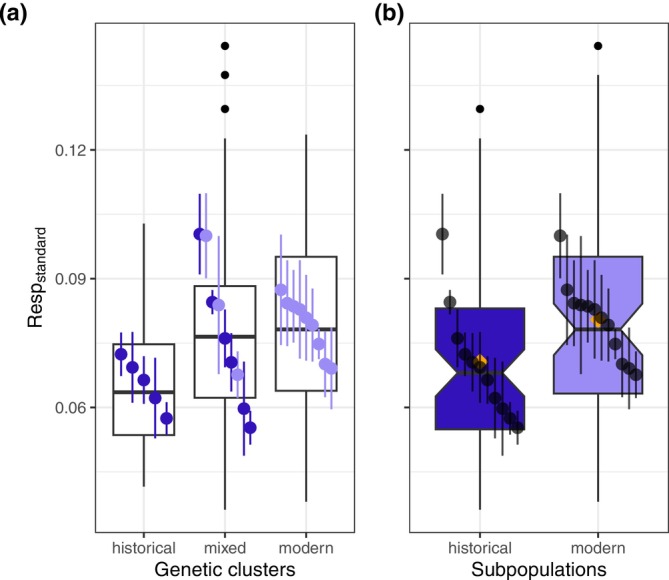
Standardized respiration rate (Resp_standard_) among genetic clusters (a) and between temporal subpopulations (b). The darker shade in both panels represents the historical subpopulation and the lighter shade the modern subpopulation. Point ranges show the mean of each clone across replicates and the standard error of the mean. The boxplot limits represent the first and third quartiles, the middle line indicating the median, whiskers are 1.5× interquartile ranges. Filled black circles above the whiskers represent outliers. In Panel b the mean values of the subpopulations are shown by orange diamonds.

We observed a trend for respiration rates to be higher in the modern cluster (historical < mixed < modern) as well as in the modern compared with the historical subpopulation (Figure 4). These differences, however, were not statistically significant, and the null models were selected by the likelihood‐ratio test in both sets of comparisons (Table 2).

**TABLE 2:** Model comparison with likelihood‐ratio test for respiration data.

	npar	AIC	BIC	logLik	Deviance	Chisq	df	p‐value
Model comparison for genetic cluster								
gen0: Resp_standard_ ~ 1 + (1|Run) + (1|Genetic Cluster:Clone)	4	−655.82	−644.17	331.91	−663.82			
gen: Resp_standard_ ~ Genetic Cluster + (1|Run) + (1|Genetic Cluster:Clone)	6	−655.08	−637.60	333.54	−667.08	3.25	2	0.197
Model comparison for subpopulation								
sub0: Resp_standard_ ~ 1 + (1|Run) + (1|Subpopulation:Clone)	4	−655.82	−644.17	331.91	−663.82			
sub: Resp_standard_ ~ Subpopulation + (1|Run) + (1|Subpopulation:Clone)	5	−656.07	−641.50	333.03	−666.07	2.24	1	0.134

### 3.5 *P*_crit_

**FIGURE 5:**
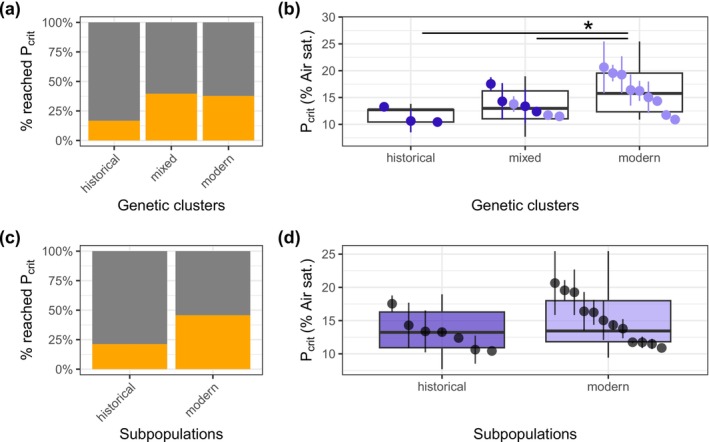
Critical oxygen limit in historical and modern subpopulations. Barplots of the percentage of replicates that reached the critical oxygen limit (*P*_crit_) and boxplots of % air saturation values of *P*_crit_ per genetic cluster (a, b) and subpopulation (c, d). Percentages that reached *P*_crit_ are shown in orange. The historical and modern subpopulation are represented by darker and lighter purple shades, respectively. Point ranges are the means per clone with SE. The boxplots were computed as in Figure 4. Horizontal bars above the boxes represent post hoc comparisons between genetic clusters (Holm method). The statistical significance of the *p* value is shown by asterisks (*< 0.05). Both comparisons share the same statistical significance.

Most individuals did not reach the critical oxygen limit (*P*_crit_) within the 6 h measurement (Figure 5a,c). This was mostly because low oxygen consumption prevented oxygen levels from approaching *P*_crit_ (see Figure S2). Notably, fewer individuals from the historical subpopulation reached *P*_crit_, emphasizing the observed trend for lower respiration rates of the historical subpopulation. The same pattern was observed for the historical genetic cluster. The Likelihood value for the full model was slightly higher for both model sets, with marginal significance when introducing subpopulations to the full model (Table 3a). The full model results show that the probability of reaching *P*_crit_ was significantly dependent upon subpopulation membership but not genetic cluster (Table 3b,c). Differences in *P*_crit_ were significant among genetic clusters (*F*_(2,43)_ = 4.72, *p* = 0.014) and the modern genetic cluster had a significantly higher *P*_crit_ than both other clusters (Figure 5b, Table S4). *P*_crit_ did not significantly differ between subpopulations (Figure 5d; *t*_(25.52)_ = −1.1, *p* = 0.28). Finally, the overall regression between weight‐adjusted respiration rates and *P*_crit_ was statistically significant, with a positive relationship between the two (adj. *R* ^2^ = 0.088, *F*_(1,44)_ = 5.39, *p* = 0.024; Figure S3), suggesting a significant association between the two variables.

**TABLE 3:** Model comparison with likelihood‐ratio test (a) and model results for binomial glmm for genetic cluster (b) and subpopulation (c) for the probability of reaching *P*_crit_. Where binom_*P*_crit_ is the success/failure of reaching *P*_crit_.

a. Model comparisons	npar	AIC	BIC	logLik	Deviance	Chisq	df	p‐value
Set 1								
null: binom_*P*_crit_ ~ 1 + (1|Run) + (1|Genetic Cluster:Clone)	3	172.29	181.03	−83.14	166.29			
*P*_crit_ gen: binom_*P*_crit_ ~ Genetic Cluster + (1|Run) + (1|Genetic Cluster:Clone)	5	172.73	187.29	−81.36	162.73	3.56	2	0.169
Set 2								
null: binom_*P*_crit_ ~ 1 + (1|Run) + (1|Subpopulation:Clone)	3	172.29	181.03	−83.14	166.29			
*P*_crit_ sub: binom_*P*_crit_ ~ Subpopulation + (1|Run) + (1|Subpopulation:Clone)	4	170.87	182.52	−81.44	162.87	3.42	1	0.065

b. Model *P*_crit_ gen: binom_*P*_crit_ ~ Genetic Cluster + (1|Run) + (1|Gentic Cluster:Clone)				
	Estimate	Std. error	*z* value	p‐value
Intercept	−1.33	0.71	−1.89	0.059
Genetic cluster mixed	1.00	0.68	1.46	0.143
Genetic cluster modern	0.16	0.98	0.17	0.868

c. Model *P*_crit_ sub: binom_*P*_crit_ ~ Subpopulation + (1|Run) + (1|Subpopulation:Clone)				
	Estimate	Std. error	*z* value	p‐value
Intercept	−1.40	0.42	−3.31	9.25e‐4
Subpopulation modern	1.15	0.56	2.07	**0.039**

## 4 Discussion

We tested the adaptation potential of an asexual *Daphnia* population in response to rapid environmental change, focusing on temperature rise and its relevance for hypoxia tolerance. For this, we applied a resurrection ecology approach to compare two temporal snapshots of this population, assessing resurrected *Daphnia* from a sediment layer dating to 2011 and contemporary clonal lineages from the population sampled in 2022. Whole genome sequencing of all clones participating in the experimental work of this study identified three genetic clusters, of which two are specific to each temporal subpopulation and a third which contains clones of both subpopulations. The genetic clusters and temporal subpopulations provide similar interpretations of the results (except in the case of *P*_crit_). We found that modern and historical *Daphnia* differ in both thermal and hypoxia tolerance, albeit at different magnitudes. Contrary to our prediction, *T*_imm_ was shorter for the modern *Daphnia* (both subpopulation and genetic cluster), a proxy that indicates tolerance to high temperatures. Additionally, we observed slightly higher respiration rates for the modern subpopulation and significantly higher *P*_crit_ for the modern genetic cluster, both indicating a lower tolerance to hypoxia. The combined pattern of low thermal and hypoxia tolerance points to the shared physiological coping mechanisms that can enhance oxygen uptake (e.g., hemoglobin increase). We discuss these results in the light of the current climate change in the study area that has significant effects on lake stratification and the lakes' oxygen environment (Hazuková et al. 2024).

Our results suggest the occurrence of phenotypic change in an asexual *Daphnia* population within a decade of environmental change among three closely related genetic clusters. The high similarity seen among the clusters (highest pairwise dissimilarity between clusters was 13.33% within the SNP set, equivalent to 0.03% divergence genome‐wide) could suggest common ancestry via long‐term molecular evolution; however, the maximum number of SNPs we found between clusters (13.33% corresponds to 48,717 SNPs) is elevated in comparison with SNPs found between diploid Japanese obligate asexual lineages of *Daphnia pulex* (~5000), estimated to have diverged molecularly during the last ~300 years based on nuclear substitutions (Ohtsuki et al. 2022). A higher SNP number could partly be explained by the higher mutation rates occurring in polyploids in comparison to diploids (Meirmans et al. 2018). Additionally, even though it is likely that polar regions including Greenland harbor only obligate parthenogenetic *Daphnia* (Weider et al. 1999; Decaestecker et al. 2009; Haileselasie 2016) and sexually reproducing *Daphnia* populations have not (yet) been found in the study area, the possibility of local clonal recruitment through sexual turnover cannot be completely excluded.

Two of the genetic clusters are specific to one of each time period, and a third has members of both periods. The most likely explanation would be an undetected, local presence of members of the modern cluster at a low frequency in the historical subpopulation, rising in frequency due to clonal selection. Alternatively, dispersal from nearby lakes cannot be excluded. In both cases, the recent change in the clonal frequencies contrasts with the results of a previous study (Dane et al. 2020) that showed the long‐term dominance of a single clone for at least the last 200 years until ~2008, identified by microsatellite markers. A final possibility is that these genetic clusters are all closely related to the dominant clone found previously, with the lower resolution of the microsatellite analysis being unable to detect them.

A lower tolerance to high temperatures, as observed in the modern clones (and more pronounced in the modern genetic cluster) while temperature in the area is rising (Saros 2019), contradicts our expectations about the direction of phenotypic change. Climate data point to increased variability in the annual spring and summer temperatures rather than a linear increase. While several of our August temperature profiles for SS4 do not show an extreme difference in surface water temperatures, earlier ice‐out is predicted to lead to a deepening of surface water mixing, resulting in a shift of the thermocline and the oxycline to greater water depths (Hazuková et al. 2024). Longer mixing periods in early ice‐out years before the onset of summer stratification might have contributed to the slightly lower temperature of the upper mixed layer in the depth profile of the earliest ice‐out year, 2019. Our data suggest a deepening of the thermocline during years of earlier ice‐out with slightly higher water temperatures in greater depths, thus possibly reducing the thermal refugium for *Daphnia*. However, this small increase in water temperature has apparently not yet affected the thermal tolerance of the SS4 population shielded by its adaptations to hypoxia. Nevertheless, our data indicate an indirect effect of warming linked to early ice‐out dates and the possible consequences for the lake ecosystem. Further warming and more frequent early ice‐out events in future years may catch up with the population's thermal tolerance, adding a direct effect and pushing it to a maladapted state.

The lower tolerance to hypoxia observed for the modern subpopulation, and especially for the modern genetic cluster, may prevent *Daphnia* from successfully grazing near or in the anoxic layer. It has been shown that *Daphnia* can utilize phototrophic purple sulfur bacteria (PSB) as a food source (Jürgens et al. 1994; Massana et al. 1994). *Daphnia's* dependence on PSB, which are a supplement to the scarce algal food sources, may be stronger in Braya Sø or other oligotrophic meromictic lakes occurring in the Kangerlussuaq area. However, according to paleolimnological pigment analysis on the lake sediment of Braya Sø, the PSB population has fluctuated in size in the past 500 years and seems to have been declining since 2000 (Dane et al. 2020; Anderson et al. submitted). In line with these data, we were unable to locate the PSB population during field sampling in August 2022, a season when the bacterial plates of PSB are typically present. The presence of dense PSB bacterial plates in August has, for example, been observed in Lake Cadagno, an alpine lake with climatic conditions similar to those in West Greenland (Tonolla et al. 2003; Storelli et al. 2024). The deepening of the oxycline observed in the 2019 and 2022 profiles might indicate an important trend occurring since 2010, with early ice‐out occurring more frequently. According to Hazuková et al. (2024), earlier ice‐out induces more prolonged spring mixing in holomictic lakes before the summer stratification, expanding the presence of oxygen deeper into the water column. Since phototrophic PSB rely on both sufficient light and anoxic conditions (Overmann 2008), this shift in the oxycline is likely to destabilize the PSB population, a situation that could be perpetuated in the following years.

The presence of fish in Braya Sø would be another reason for the *Daphnia* to descend closer to the anoxic zone (Larsson and Lampert 2011). However, bigger *Daphnia* species like the polyploid Arctic *D. pulicaria* and fish generally do not co‐occur in lakes of West Greenland (Jeppesen 2017). Hence, a reduced or absent PSB layer could be a major reason for tolerance to hypoxia no longer conferring an important benefit. One central mechanism for adaptation to hypoxia is through elevated hemoglobin (Hb) production and oxygen affinity (Paul, Zeis, et al. 2004; Seidl et al. 2005). Other mechanisms are changes in carbohydrate‐degrading enzymes and a decrease in oxygen consumption and *P*_crit_ (Seidl et al. 2005; Zeis et al. 2009). These adaptations involving elevated protein synthesis may confer costs (Pirow et al. 2001), and therefore the relaxation of this selection pressure could explain a lower hypoxia tolerance in the modern subpopulation.

Finally, a similar trend for the two measured traits (low tolerance to hypoxia combined with low thermal tolerance and vice versa) suggests a relationship with the physiological connection of the two traits as explained by the oxygen‐limited thermal tolerance hypothesis (Pörtner 2002). This hypothesis posits that the upper thermal limit of an organism is set by oxygen limitation and transportation capacity and has found more support in water‐breathing arthropods in comparison to air‐breathers (Verberk et al. 2016). This has also been observed in *Daphnia*, where elevated oxygen demand at higher temperatures caused an increase in Hb production similar to hypoxic conditions and rising oxygen consumption (Fox and Phear 1953; Paul, Lamkemeyer, et al. 2004). Higher gene expression of hemoglobin has been measured as a reaction to both hypoxia and raised temperature (Lamkemeyer et al. 2003; Becker et al. 2011; Zeis 2020), while increased Hb levels were correlated with longer *T*_imm_ (Yampolsky et al. 2014). This common mechanism suggests that a shift in hypoxia tolerance in response to the environment would also affect thermal tolerance.

One caveat of this study is the use of single proxies for both thermal and hypoxia tolerance, possibly obscuring more fine‐tuned changes in the traits measured. While our *T*_imm_, respiration, and *P*_crit_ measurements demonstrate differences between subpopulations and genetic clusters, a better resolution of thermal responses, for example, utilizing thermal performance curves (Alruiz et al. 2023) as well as responses to various degrees of hypoxia could help disentangle the physiological responses to warming and to changes in the oxygen environment of the lake.

In conclusion, we observed phenotypic change over a short time period in an asexual population of *Daphnia*. The direction of this change was, however, contrary to our expectation. Even though temperature rise is one of the most prevalent factors of global change, our findings suggest that at least until now temperature was not the driving stress factor for this population. In contrast, the destabilization of the PSB population and thus a reduced demand for hypoxia tolerance may have outbalanced the costs of hypoxia adaptation such as elevated hemoglobin production, leading simultaneously to a lower thermal tolerance. The consequences of this phenotypic change could increase the population's vulnerability to future warming. This finding further demonstrates the complexity of the impacts of environmental change in ecosystems and how local adaptations to unique environmental factors affect population responses. In addition, we found three genetic clusters in this asexual population that may have resulted from molecular evolution. Whether these genetic clusters are only found in this lake or have dispersed from other regional lakes is unknown.

Our findings pose the question of whether the relatively small genome‐wide differences among the genomic clusters could explain the associated phenotypic divergence. In future research, it will be important to disentangle the molecular underpinnings, in particular regarding gene expression profiles, epigenetic modifications, or ploidy‐related effects, which could underlie the observed phenotypes. The presence of such mechanisms could be crucial for asexually reproducing animals to keep up with the contemporary fast‐paced environmental change, ultimately allowing their survival.

## Author Contributions

**Athina Karapli‐Petritsopoulou:** conceptualization, formal analysis, investigation, methodology, visualization, writing – original draft, writing – review and editing. **Jasmin Josephine Heckelmann:** investigation, methodology, writing – review and editing. **Dörthe Becker:** conceptualization, methodology, writing – review and editing. **N. John Anderson:** conceptualization, formal analysis, resources, visualization, writing – review and editing. **Dagmar Frisch:** conceptualization, formal analysis, funding acquisition, investigation, methodology, project administration, resources, supervision, visualization, writing – original draft, writing – review and editing.

## Conflicts of Interest

The authors declare no conflicts of interest.

## Supporting information


Data S1.

## Data Availability

The data and code that support the findings of this study are openly available in Zenodo at http://doi.org/10.5281/zenodo.14946376. Fastq files of DNA sequences included here are available at GenBank under Bioproject PRJNA1147697 with accession numbers SRR32522081—SRR32522099 and SRR30223773—SRR30223776.
