# Supplementary material for: Altered Phenotypic Responses of Asexual Arctic Daphnia After 10 Years of Rapid Climate Change
Source: Glob Chang Biol. 2025 Mar 18;31(3):e70119. doi: 10.1111/gcb.70119 (PMC11915199; doi:10.1111/gcb.70119)
Supplement: Supplementary file 1 — Data S1. [file GCB-31-e70119-s001.pdf]

Supplementary Material

Altered phenotypic responses of asexual Arctic *Daphnia* after 10 years of rapid climate change

Authors:

Athina Karapli-Petritsopoulou[1,2], Jasmin Josephine Heckelmann[1], Dörthe Becker[3], N. John Anderson[4], Dagmar Frisch[1]

Affiliations:

[1] Department of Evolutionary and Integrative Ecology, Leibniz Institute of Freshwater Ecology and Inland Fisheries (IGB), Berlin, Germany

[2] Department of Biology, Chemistry, Pharmacy, Institute of Biology, Freie Universität Berlin, Berlin, Germany

[3] Naturschutzstation Niederrhein (NABU), Kleve, Germany

[4] Department of Geography & Environment, Loughborough University, Loughborough, UK

Supplementary Figures

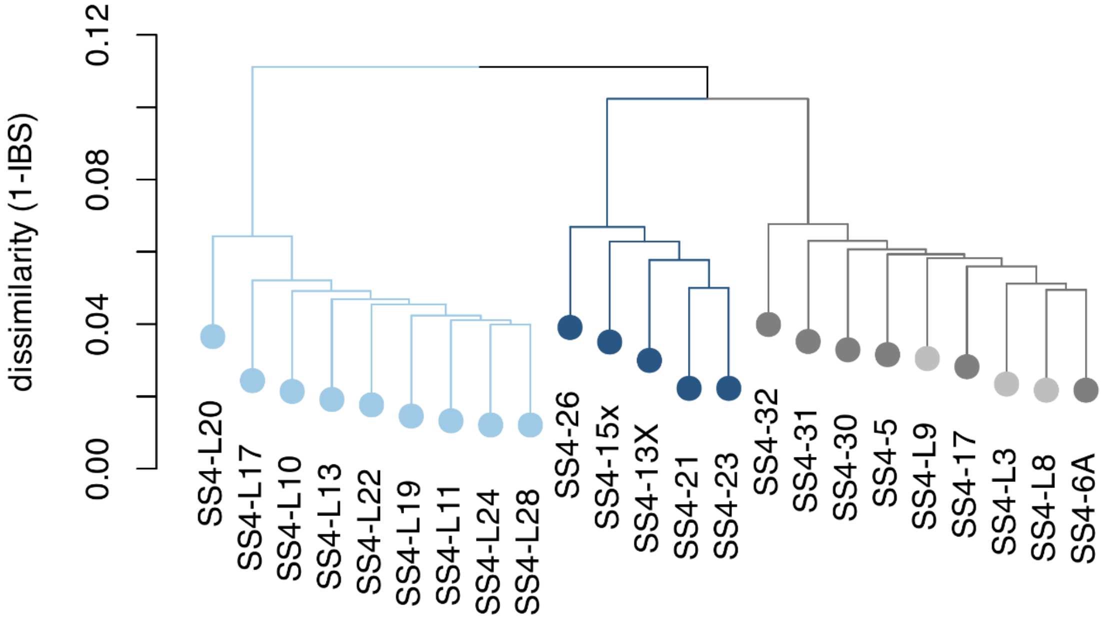

Figure S1. Dendrogram of genetic clusters of clonal lineages based on identity-by-state (IBS) labeled for clone. Dark and light shades represent historical and modern clones respectively. The historical cluster is shown in dark blue, the modern cluster in light blue and the mixed cluster in grey.

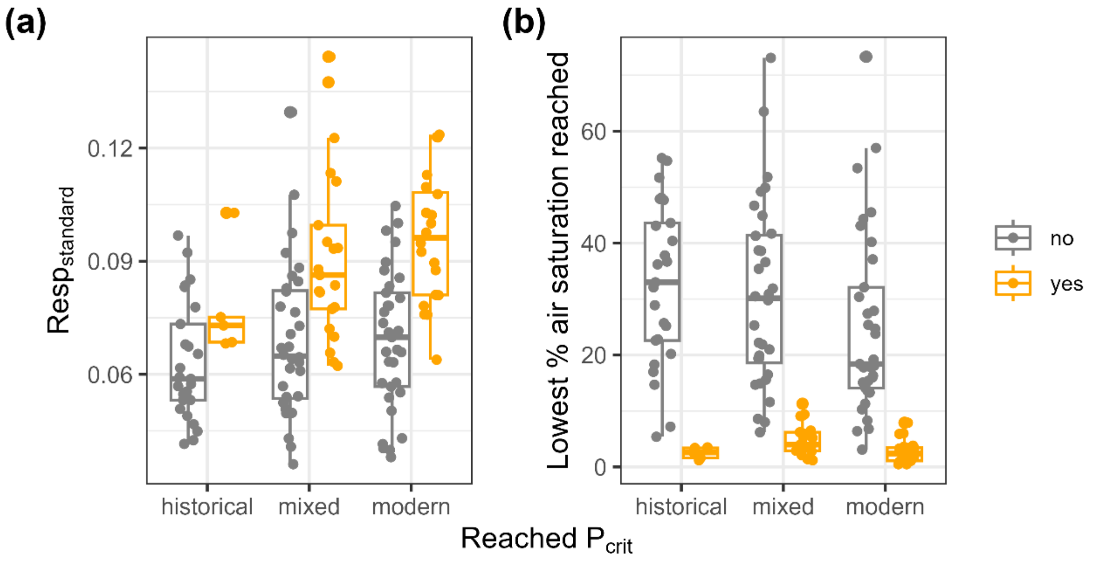

Figure S2. $Resp_{standard}$ (**a**) and lowest % air saturation value reached at the end of six hours (**b**) per genetic cluster according to whether individuals reached P_{crit}. Individuals where P_{crit} was successfully measured are coloured with orange, while grey points show non-obtained P_{crit} data.

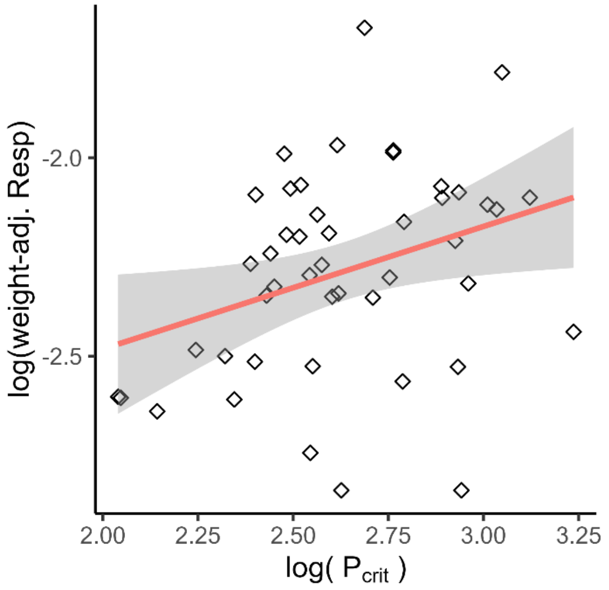

Figure S3. Linear regression between weight-adjusted respiration rates and P_{crit} (model: log(P_{crit}) ~ log(weight_adj_rates).

Supplementary Tables

Table S1. Lead-210 dating of sediment core where *Daphnia* were hatched from.

Top of Interval	Base of Interval	Mid of Interval	Total 210Pb	Error of Total Pb	Cum. Dry Mass	Unsup. 210Pb	Error of Unsup Pb	Age: Base of Int.	Error of Age	Date: Base A.D.	Date: Mid A.D.	Sediment DMAR	Error of DMAR
(cm)	(cm)	(cm)	(pCi/g)	(±s.d.)	(g/cm2)	(pCi/g)	(±s.d.)	(yr)	(±s.d.)			(g/cm2 yr)	(±s.d.)
0	0.5	0.25	10.520	0.425	0.032	10.294	0.425	3.96	0.76	2018.4	2020.4	0.0081	0.0003
0.5	1	0.75	9.311	0.274	0.056	9.085	0.274	6.92	0.80	2015.4	2016.9	0.0082	0.0003
1	1.5	1.25	8.395	0.309	0.096	8.169	0.309	11.85	0.86	2010.5	2013.0	0.0081	0.0003
1.5	2	1.75	9.362	0.257	0.137	9.137	0.257	18.71	0.97	2003.6	2007.1	0.0060	0.0002
2	2.5	2.25	5.849	0.189	0.191	5.624	0.189	25.54	1.10	1996.8	2000.2	0.0079	0.0003
2.5	3	2.75	3.602	0.124	0.287	3.377	0.125	34.92	1.34	1987.4	1992.1	0.0102	0.0005
3	3.5	3.25	3.066	0.111	0.366	2.841	0.112	43.52	1.66	1978.8	1983.1	0.0092	0.0005
3.5	4	3.75	2.094	0.047	0.437	1.869	0.049	49.90	1.98	1972.4	1975.6	0.0111	0.0007
4	4.5	4.25	1.570	0.052	0.562	1.345	0.054	60.54	2.66	1961.8	1967.1	0.0118	0.0009
4.5	5	4.75	1.019	0.029	0.735	0.794	0.032	72.92	3.83	1949.4	1955.6	0.0140	0.0015
5	5.5	5.25	0.711	0.022	0.893	0.486	0.026	82.63	5.12	1939.7	1944.6	0.0162	0.0023
5.5	6	5.75	0.541	0.018	1.039	0.316	0.023	90.35	6.47	1932.0	1935.9	0.0190	0.0036
6	6.5	6.25	0.514	0.017	1.195	0.288	0.022	100.23	8.74	1922.1	1927.1	0.0158	0.0038
6.5	7	6.75	0.346	0.011	1.351	0.121	0.018	105.47	10.25	1916.9	1919.5	0.0297	0.0096
7	7.5	7.25	0.443	0.016	1.511	0.218	0.021	118.33	15.21	1904.0	1910.5	0.0125	0.0050
7.5	8	7.75	0.380	0.012	1.662	0.154	0.019	131.01	22.50	1891.3	1897.7	0.0119	0.0070
8	8.5	8.25	0.336	0.010	1.799	0.111	0.017	143.27	32.86	1879.1	1885.2	0.0112	0.0096

Table S2. Pair-wise dissimilarity matrix (1-IBS) between clones. Clone labels are coloured according to genetic cluster and subpopulation membership (see colour code in Figure S1).

	SS4-L20	SS4-L17	SS4-L10	SS4-L13	SS4-L22	SS4-L19	SS4-L11	SS4-L24	SS4-L28	SS4-32	SS4-31	SS4-30	SS4-5	SS4-L9	SS4-17	SS4-L3	SS4-L8	SS4-6A	SS4-26	SS4-15x	SS4-13X	SS4-21	SS4-23
SS4-L20	0	0.06922	0.06734	0.06567	0.06503	0.06257	0.06241	0.06068	0.06173	0.13333	0.13007	0.12857	0.12787	0.1279	0.12673	0.12343	0.12319	0.12162	0.12488	0.12346	0.12126	0.1092	0.11942
SS4-L17	0.06922	0	0.05577	0.05462	0.05374	0.05127	0.05055	0.04894	0.05017	0.12342	0.12053	0.11869	0.11801	0.11794	0.11699	0.11325	0.11279	0.11117	0.11521	0.11351	0.11101	0.09848	0.10937
SS4-L10	0.06734	0.05577	0	0.05206	0.05113	0.04877	0.04854	0.04669	0.0478	0.12136	0.11798	0.11655	0.11574	0.11576	0.11494	0.11101	0.11066	0.10901	0.11276	0.11114	0.10909	0.09633	0.10701
SS4-L13	0.06567	0.05462	0.05206	0	0.04993	0.04707	0.0468	0.04483	0.04601	0.12054	0.11709	0.11521	0.11447	0.11506	0.11346	0.10976	0.10932	0.10788	0.11201	0.10998	0.10782	0.09482	0.10566
SS4-L22	0.06503	0.05374	0.05113	0.04993	0	0.04622	0.0461	0.04411	0.04535	0.11982	0.11657	0.11475	0.11386	0.11404	0.11279	0.10905	0.10861	0.10697	0.1114	0.1093	0.10731	0.09432	0.10504
SS4-L19	0.06257	0.05127	0.04877	0.04707	0.04622	0	0.04331	0.04128	0.04247	0.1176	0.11433	0.11243	0.11178	0.11179	0.11084	0.10671	0.10645	0.10455	0.10927	0.10712	0.10467	0.0914	0.10268
SS4-L11	0.06241	0.05055	0.04854	0.0468	0.0461	0.04331	0	0.04055	0.04152	0.11741	0.1137	0.11177	0.11111	0.11092	0.10984	0.10646	0.10577	0.10403	0.10889	0.10711	0.10457	0.09125	0.10283
SS4-L24	0.06068	0.04894	0.04669	0.04483	0.04411	0.04128	0.04055	0	0.03987	0.11574	0.11195	0.10981	0.10936	0.10925	0.10807	0.10445	0.10396	0.10235	0.10723	0.10563	0.10306	0.08912	0.10113
SS4-L28	0.06173	0.05017	0.0478	0.04601	0.04535	0.04247	0.04152	0.03987	0	0.11675	0.113	0.111	0.11019	0.11023	0.10916	0.10569	0.10538	0.10355	0.10813	0.1063	0.10415	0.0902	0.10214
SS4-32	0.13333	0.12342	0.12136	0.12054	0.11982	0.1176	0.11741	0.11574	0.11675	0	0.07247	0.07044	0.06928	0.06963	0.06891	0.06432	0.06402	0.06242	0.11486	0.11241	0.11064	0.09915	0.10835
SS4-31	0.13007	0.12053	0.11798	0.11709	0.11657	0.11433	0.1137	0.11195	0.113	0.07247	0	0.06638	0.06544	0.06529	0.06433	0.06067	0.06009	0.05872	0.11105	0.10958	0.10741	0.09498	0.1056
SS4-30	0.12857	0.11869	0.11655	0.11521	0.11475	0.11243	0.11177	0.10981	0.111	0.07044	0.06638	0	0.06363	0.06384	0.06265	0.05887	0.05818	0.05691	0.1096	0.10792	0.10567	0.09304	0.10398
SS4-5	0.12787	0.11801	0.11574	0.11447	0.11386	0.11178	0.11111	0.10936	0.11019	0.06928	0.06544	0.06363	0	0.06304	0.06208	0.05812	0.05748	0.05595	0.10855	0.10689	0.10466	0.0923	0.10244
SS4-L9	0.1279	0.11794	0.11576	0.11506	0.11404	0.11179	0.11092	0.10925	0.11023	0.06963	0.06529	0.06384	0.06304	0	0.06183	0.05784	0.05747	0.05589	0.10901	0.10717	0.10492	0.09223	0.10315
SS4-17	0.12673	0.11699	0.11494	0.11346	0.11279	0.11084	0.10984	0.10807	0.10916	0.06891	0.06433	0.06265	0.06208	0.06183	0	0.05688	0.05646	0.05465	0.10796	0.10597	0.104	0.09149	0.10206
SS4-L3	0.12343	0.11325	0.11101	0.10976	0.10905	0.10671	0.10646	0.10445	0.10569	0.06432	0.06067	0.05887	0.05812	0.05784	0.05688	0	0.0522	0.05021	0.10441	0.10233	0.10037	0.08752	0.09811
SS4-L8	0.12319	0.11279	0.11066	0.10932	0.10861	0.10645	0.10577	0.10396	0.10538	0.06402	0.06009	0.05818	0.05748	0.05747	0.05646	0.0522	0	0.0495	0.10402	0.10196	0.09987	0.08698	0.09781
SS4-6A	0.12162	0.11117	0.10901	0.10788	0.10697	0.10455	0.10403	0.10235	0.10355	0.06242	0.05872	0.05691	0.05595	0.05589	0.05465	0.05021	0.0495	0	0.10208	0.10045	0.09823	0.08538	0.09626
SS4-26	0.12488	0.11521	0.11276	0.11201	0.1114	0.10927	0.10889	0.10723	0.10813	0.11486	0.11105	0.1096	0.10855	0.10901	0.10796	0.10441	0.10402	0.10208	0	0.07249	0.06994	0.05697	0.06804
SS4-15x	0.12346	0.11351	0.11114	0.10998	0.1093	0.10712	0.10711	0.10563	0.1063	0.11241	0.10958	0.10792	0.10689	0.10717	0.10597	0.10233	0.10196	0.10045	0.07249	0	0.06813	0.05514	0.06521
SS4-13X	0.12126	0.11101	0.10909	0.10782	0.10731	0.10467	0.10457	0.10306	0.10415	0.11064	0.10741	0.10567	0.10466	0.10492	0.104	0.10037	0.09987	0.09823	0.06994	0.06813	0	0.0523	0.06319
SS4-21	0.1092	0.09848	0.09633	0.09482	0.09432	0.0914	0.09125	0.08912	0.0902	0.09915	0.09498	0.09304	0.0923	0.09223	0.09149	0.08752	0.08698	0.08538	0.05697	0.05514	0.0523	0	0.05002
SS4-23	0.11942	0.10937	0.10701	0.10566	0.10504	0.10268	0.10283	0.10113	0.10214	0.10835	0.1056	0.10398	0.10244	0.10315	0.10206	0.09811	0.09781	0.09626	0.06804	0.06521	0.06319	0.05002	0

Table S3. Post-hoc test among genetic clusters for gen1(T_{imm} ~ Genetic Cluster + (1|Run)) for T_{imm} data using Holm method.

Gen. cluster comparison	Estimate	Std. Error	z value	Pr(>|z|)
mixed - historical	-158.3	250.5	-0.63	0.527
modern -historical	-1271.1	255.7	-4.97	**1.33E-06**
modern - mixed	-1112.8	62.8	-6.84	**2.45E-11**

Table S4. Post-hoc test among genetic clusters (for ANOVA model Pcrit ~ genetic cluster) for P_{crit} using Holm method.

Gen. cluster comparison	Estimate	Std. Error	t value	Pr(>|t|)
mixed - historical	1.72	1.86	0.93	0.621
modern -historical	4.65	1.87	2.49	**0.042**
modern - mixed	2.93	1.17	2.51	**0.040**
